# Supplementary material for: Visualization of stem cell activity in pancreatic cancer expansion by direct lineage tracing with live imaging
Source: eLife. 2021 Jan 4;10:e55117. doi: 10.7554/eLife.55117 (PMC7800378; doi:10.7554/eLife.55117)
Supplement: Figure 1—source data 1. [file elife-55117-fig1-data1.docx]

**Figure 1-Source Data 1**

|  | Dclk1 | CK19 |  |  |  |
| --- | --- | --- | --- | --- | --- |
| KF_01 | 745 | 12104 | 6.154990 |  |  |
| KF_02 | 330 | 3997 | 8.256192 |  |  |
| KF_03 | 108 | 2886 | 3.742204 |  |  |
| KF_04 | 22 | 461 | 4.772234 |  |  |
| KF_05 | 205 | 2969 | 6.904682 | AVG | 6.539006 |
| KF_06 | 23 | 845 | 2.721893 | SD | 3.497400 |
| KF_07 | 619 | 4682 | 13.220846 | SE | 1.321893 |
