# Supplementary material for: Visualization of stem cell activity in pancreatic cancer expansion by direct lineage tracing with live imaging
Source: eLife. 2021 Jan 4;10:e55117. doi: 10.7554/eLife.55117 (PMC7800378; doi:10.7554/eLife.55117)
Supplement: Figure 1—source data 2. [file elife-55117-fig1-data2.docx]

**Figure 1-Source Data 2**

|  | Dclk1 | CK19 |  |  |  |
| --- | --- | --- | --- | --- | --- |
| KPF_01 | 25 | 7172 | 0.348578 |  |  |
| KPF_02 | 15 | 9215 | 0.162778 |  |  |
| KPF_03 | 26 | 12097 | 0.214929 |  |  |
| KPF_04 | 9 | 6009 | 0.149775 |  |  |
| KPF_05 | 15 | 8759 | 0.171252 |  |  |
| KPF_06 | 7 | 9868 | 0.070936 | AVG | 0.173231 |
| KPF_07 | 6 | 4578 | 0.131062 | SD | 0.081712 |
| KPF_08 | 10 | 7324 | 0.136537 | SE | 0.028889 |
