# Supplementary material for: Visualization of stem cell activity in pancreatic cancer expansion by direct lineage tracing with live imaging
Source: eLife. 2021 Jan 4;10:e55117. doi: 10.7554/eLife.55117 (PMC7800378; doi:10.7554/eLife.55117)
Supplement: Figure 1—source data 3. [file elife-55117-fig1-data3.docx]

**Figure 1-Source Data 3**

|  | Dclk1 | Total |  |  |  |
| --- | --- | --- | --- | --- | --- |
| hPDAC_01 | 14 | 15713 | 0.000891 |  |  |
| hPDAC_02 | 3 | 21808 | 0.000138 |  |  |
| hPDAC_03 | 31 | 25826 | 0.001200 |  |  |
| hPDAC_04 | 25 | 13708 | 0.001824 |  |  |
| hPDAC_05 | 3 | 18057 | 0.000166 | AVG | 0.000974 |
| hPDAC_06 | 7 | 8487 | 0.000825 | SD | 0.000683 |
| hPDAC_07 | 35 | 19722 | 0.001775 | SE | 0.000258 |
