# Supplementary material for: Visualization of stem cell activity in pancreatic cancer expansion by direct lineage tracing with live imaging
Source: eLife. 2021 Jan 4;10:e55117. doi: 10.7554/eLife.55117 (PMC7800378; doi:10.7554/eLife.55117)
Supplement: Figure 1—source data 4. [file elife-55117-fig1-data4.docx]

**Figure 1-Source Data 4**

|  | Epcam^+^ cells | PanIN cells | Ratio |
| --- | --- | --- | --- |
| PanIN_01 | 2936 | 4034 | 0.727814 |
| PanIN_02 | 2283 | 3340 | 0.683533 |
| PanIN_03 | 1277 | 1793 | 0.712214 |
| PanIN_04 | 806 | 1184 | 0.680743 |
| PanIN_05 | 1673 | 2395 | 0.698539 |
|  |  | AVG | 0.700569 |
|  |  | SD | 0.019782 |
|  |  | SE | 0.008847 |

|  | CD44^+^ cells | PanIN cells | Ratio |
| --- | --- | --- | --- |
| PanIN_01 | 1837 | 2757 | 0.666304 |
| PanIN_02 | 1319 | 2154 | 0.612349 |
| PanIN_03 | 2698 | 4019 | 0.671311 |
| PanIN_04 | 1085 | 1683 | 0.644682 |
| PanIN_05 | 1953 | 2945 | 0.663158 |
|  |  | AVG | 0.651561 |
|  |  | SD | 0.024118 |
|  |  | SE | 0.010786 |

|  | CD24^+^ cells | PanIN cells | Ratio |
| --- | --- | --- | --- |
| PanIN_01 | 550 | 3336 | 0.164868 |
| PanIN_02 | 409 | 2626 | 0.155750 |
| PanIN_03 | 688 | 3574 | 0.192501 |
| PanIN_04 | 367 | 2072 | 0.177124 |
| PanIN_05 | 224 | 1657 | 0.135184 |
|  |  | AVG | 0.165085 |
|  |  | SD | 0.021667 |
|  |  | SE | 0.009690 |

|  | Aldh1a1^+^ cells | PanIN cells | Ratio |
| --- | --- | --- | --- |
| PanIN_01 | 120 | 2482 | 0.048348 |
| PanIN_02 | 157 | 1797 | 0.087368 |
| PanIN_03 | 162 | 3290 | 0.049240 |
| PanIN_04 | 174 | 2272 | 0.076585 |
| PanIN_05 | 125 | 1957 | 0.063873 |
|  |  | AVG | 0.065083 |
|  |  | SD | 0.017040 |
|  |  | SE | 0.007620 |
