# Supplementary material for: Visualization of stem cell activity in pancreatic cancer expansion by direct lineage tracing with live imaging
Source: eLife. 2021 Jan 4;10:e55117. doi: 10.7554/eLife.55117 (PMC7800378; doi:10.7554/eLife.55117)
Supplement: Figure 1—source data 5. [file elife-55117-fig1-data5.docx]

**Figure 1-Source Data 5**

|  | Epcam^+^ cells | PDAC cells | Ratio |
| --- | --- | --- | --- |
| PDAC_01 | 3109 | 4359 | 0.713237 |
| PDAC_02 | 2790 | 3923 | 0.711190 |
| PDAC_03 | 3111 | 4482 | 0.694110 |
| PDAC_04 | 2503 | 3433 | 0.729100 |
| PDAC_05 | 1630 | 2259 | 0.721558 |
|  |  | AVG | 0.713839 |
|  |  | SD | 0.013125 |
|  |  | SE | 0.005870 |

|  | CD44^+^ cells | PDAC cells | Ratio |
| --- | --- | --- | --- |
| PDAC_01 | 2505 | 3725 | 0.672483 |
| PDAC_02 | 2795 | 4112 | 0.679718 |
| PDAC_03 | 1697 | 2486 | 0.682623 |
| PDAC_04 | 2265 | 3267 | 0.693297 |
| PDAC_05 | 1750 | 2399 | 0.729471 |
|  |  | AVG | 0.691518 |
|  |  | SD | 0.022496 |
|  |  | SE | 0.010060 |

|  | CD24^+^ cells | PDAC cells | Ratio |
| --- | --- | --- | --- |
| PDAC_01 | 466 | 2284 | 0.204028 |
| PDAC_02 | 250 | 1568 | 0.159439 |
| PDAC_03 | 613 | 3032 | 0.202177 |
| PDAC_04 | 410 | 2535 | 0.161736 |
| PDAC_05 | 270 | 1817 | 0.148597 |
|  |  | AVG | 0.175195 |
|  |  | SD | 0.025963 |
|  |  | SE | 0.011611 |

|  | Aldh1a1^+^ cells | PDAC cells | Ratio |
| --- | --- | --- | --- |
| PDAC_01 | 23 | 3073 | 0.007485 |
| PDAC_02 | 36 | 2872 | 0.012535 |
| PDAC_03 | 52 | 2040 | 0.025490 |
| PDAC_04 | 47 | 2961 | 0.015873 |
| PDAC_05 | 51 | 2460 | 0.020732 |
|  |  | AVG | 0.016423 |
|  |  | SD | 0.007002 |
|  |  | SE | 0.003131 |
