# Supplementary material for: Visualization of stem cell activity in pancreatic cancer expansion by direct lineage tracing with live imaging
Source: eLife. 2021 Jan 4;10:e55117. doi: 10.7554/eLife.55117 (PMC7800378; doi:10.7554/eLife.55117)
Supplement: Figure 2—source data 1. [file elife-55117-fig2-data1.docx]

**Figure 2-Source Data 1**

| day 0 (cells) | EGFP^+^ cells | PanIN cells | %EGFP^+^ cells | day 28 (cells) | EGFP^+^ cells | PanIN cells | %EGFP^+^ cells |
| --- | --- | --- | --- | --- | --- | --- | --- |
| PanIN 0_1 | 745 | 12104 | 0.061550 | PanIN 28_1 | 4261 | 9983 | 0.426826 |
| PanIN 0_2 | 330 | 3997 | 0.082562 | PanIN 28_2 | 979 | 3086 | 0.317239 |
| PanIN 0_3 | 108 | 2886 | 0.037422 | PanIN 28_3 | 2394 | 7206 | 0.332223 |
| PanIN 0_4 | 22 | 461 | 0.047722 | PanIN 28_4 | 3467 | 8087 | 0.428713 |
| PanIN 0_5 | 205 | 2969 | 0.069047 | PanIN 28_5 | 555 | 1570 | 0.353503 |
| PanIN 0_6 | 23 | 845 | 0.027219 | PanIN 28_6 | 1392 | 4272 | 0.325843 |
|  |  |  |  | PanIN 28_7 | 635 | 2177 | 0.291686 |
|  |  | AVG | 0.054254 |  |  | AVG | 0.353719 |
|  |  | SD | 0.020641 |  |  | SD | 0.053831 |
|  |  | SE | 0.008427 |  |  | SE | 0.020346 |
|  |  |  |  |  | F TEST | | 0.052736 |
|  |  |  |  |  | T TEST | | 6.08E-08 |
