# Supplementary material for: Visualization of stem cell activity in pancreatic cancer expansion by direct lineage tracing with live imaging
Source: eLife. 2021 Jan 4;10:e55117. doi: 10.7554/eLife.55117 (PMC7800378; doi:10.7554/eLife.55117)
Supplement: Figure 3—source data 1. [file elife-55117-fig3-data1.docx]

**Figure 3-Source Data 1**

| day 0 (pixel) | EGFP^+^ area | CK19^+^ area | %EGFP^+^ area | day 14 (pixel) | EGFP^+^ area | CK19^+^ area | %EGFP^+^ area |
| --- | --- | --- | --- | --- | --- | --- | --- |
| PDAC 0_1 | 11589 | 34155521 | 0.000339 | PDAC 14_1 | 5501769 | 16028209 | 0.343255 |
| PDAC 0_2 | 3235 | 15351315 | 0.000211 | PDAC 14_2 | 14018204 | 20506349 | 0.683603 |
| PDAC 0_3 | 244 | 523006 | 0.000467 | PDAC 14_3 | 4469812 | 6919977 | 0.645929 |
| PDAC 0_4 | 15829 | 36229803 | 0.000437 | PDAC 14_4 | 3731890 | 8028855 | 0.464810 |
| PDAC 0_5 | 2682 | 6431424 | 0.000417 | PDAC 14_5 | 12249082 | 22448083 | 0.545663 |
| PDAC 0_6 | 2834 | 5412902 | 0.000524 | PDAC 14_6 | 5397773 | 9130176 | 0.591201 |
|  |  | AVG | 0.000399 |  |  | AVG | 0.545744 |
|  |  | SD | 0.000110 |  |  | SD | 0.125428 |
|  |  | SE | 0.000045 |  |  | SE | 0.051206 |
|  |  |  |  |  | F TEST | | 5.72E-15 |
|  |  |  |  |  | T TEST | | 0.000126 |
