# Supplementary material for: Visualization of stem cell activity in pancreatic cancer expansion by direct lineage tracing with live imaging
Source: eLife. 2021 Jan 4;10:e55117. doi: 10.7554/eLife.55117 (PMC7800378; doi:10.7554/eLife.55117)
Supplement: Figure 3—figure supplement 1—source data 1. — The number of EGFP+ cells in Dclk1− cells of pancreatic epithelium in DRF, DRKF, and DRKPF mice with immunofluorescent staining. [file elife-55117-fig3-figsupp1-data1.docx]

**Figure 3-figure supplement 1-Source Data 1**

| day 1(cells) | Dclk1^-^ | GFP^+^/Dclk1^-^ |  | day 3(cells) | Dclk1^-^ | GFP^+^/Dclk1^-^ |  |
| --- | --- | --- | --- | --- | --- | --- | --- |
| DRF 1_1 | 5953 | 0 | 0.000000 | DRF 3_1 | 5560 | 0 | 0.000000 |
| DRF 1_2 | 5580 | 1 | 0.017921 | DRF 3_2 | 5320 | 1 | 0.018797 |
| DRF 1_3 | 5911 | 1 | 0.016918 | DRF 3_3 | 5958 | 0 | 0.000000 |
| DRF 1_4 | 5158 | 0 | 0.000000 | DRF 3_4 | 5938 | 0 | 0.000000 |
| DRF 1_5 | 5899 | 0 | 0.000000 | DRF 3_5 | 6140 | 0 | 0.000000 |
|  |  |  |  | DRF 3_6 | 5680 | 1 | 0.017606 |
|  |  | AVG | 0.006968 |  |  | AVG | 0.006067 |
|  |  | SD | 0.009548 |  |  | SD | 0.009407 |
|  |  | SE | 0.004270 |  |  | SE | 0.003840 |

| day 1(cells) | Dclk1^-^ | GFP^+^/Dclk1^-^ |  | day 3(cells) | Dclk1^-^ | GFP^+^/Dclk1^-^ |  |
| --- | --- | --- | --- | --- | --- | --- | --- |
| DRKF 1_1 | 5869 | 0 | 0.000000 | DRKF 3_1 | 6206 | 0 | 0.000000 |
| DRKF 1_2 | 5963 | 1 | 0.016770 | DRKF 3_2 | 6049 | 0 | 0.000000 |
| DRKF 1_3 | 5542 | 0 | 0.000000 | DRKF 3_3 | 5609 | 1 | 0.017828 |
| DRKF 1_4 | 5669 | 0 | 0.000000 | DRKF 3_4 | 6070 | 1 | 0.016474 |
| DRKF 1_5 | 5702 | 1 | 0.017538 | DRKF 3_5 | 5739 | 0 | 0.000000 |
|  |  |  |  | DRKF 3_6 | 5739 | 0 | 0.000000 |
|  |  | AVG | 0.006862 |  |  | AVG | 0.005717 |
|  |  | SD | 0.009399 |  |  | SD | 0.008867 |
|  |  | SE | 0.004204 |  |  | SE | 0.005120 |

| day 1(cells) | Dclk1^-^ | GFP^+^/Dclk1^-^ |  | day 3 (cells) | Dclk1^-^ | GFP^+^/Dclk1^-^ |  |
| --- | --- | --- | --- | --- | --- | --- | --- |
| DRKPF 1_1 | 6078 | 0 | 0.000000 | DRKPF 3_1 | 5722 | 0 | 0.000000 |
| DRKPF 1_2 | 5967 | 0 | 0.000000 | DRKPF 3_2 | 4059 | 0 | 0.000000 |
| DRKPF 1_3 | 5485 | 2 | 0.036463 | DRKPF 3_3 | 5201 | 0 | 0.000000 |
| DRKPF 1_4 | 5892 | 0 | 0.000000 | DRKPF 3_4 | 5467 | 2 | 0.036583 |
| DRKPF 1_5 | 6133 | 0 | 0.000000 | DRKPF 3_5 | 5709 | 0 | 0.000000 |
| DRKPF 1_6 | 5936 | 0 | 0.000000 | DRKPF 3_6 | 5632 | 0 | 0.000000 |
|  |  | AVG | 0.006077 |  |  | AVG | 0.006097 |
|  |  | SD | 0.014886 |  |  | SD | 0.014935 |
|  |  | SE | 0.006657 |  |  | SE | 0.006097 |
