# Supplementary material for: Visualization of stem cell activity in pancreatic cancer expansion by direct lineage tracing with live imaging
Source: eLife. 2021 Jan 4;10:e55117. doi: 10.7554/eLife.55117 (PMC7800378; doi:10.7554/eLife.55117)
Supplement: Figure 3—figure supplement 2—source data 1. — The number of EGFP+ cells before (day 0) and 3 days after 4-OHT administration (day 3). [file elife-55117-fig3-figsupp2-data1.docx]

**Figure 3-figure supplement 2-Source Data 1**

| day 0 (cells) | EGFP^+^ cells | PDAC cells | %EGFP^+^ cells | day 3 (cells) | EGFP^+^ cells | PDAC cells | %EGFP^+^ cells |
| --- | --- | --- | --- | --- | --- | --- | --- |
| SPH 0_1 | 10 | 963 | 0.010384 | SPH 3_1 | 339 | 604 | 0.561258 |
| SPH 0_2 | 12 | 839 | 0.014303 | SPH 3_2 | 445 | 833 | 0.534214 |
| SPH 0_3 | 11 | 1292 | 0.000851 | SPH 3_3 | 441 | 664 | 0.664157 |
| SPH 0_4 | 10 | 957 | 0.010449 | SPH 3_4 | 255 | 683 | 0.373353 |
| SPH 0_5 | 10 | 1125 | 0.008899 | SPH 3_5 | 226 | 523 | 0.432122 |
|  |  | AVG | 0.010508 |  |  | AVG | 0.513021 |
|  |  | SD | 0.002292 |  |  | SD | 0.113655 |
|  |  | SE | 0.001025 |  |  | SE | 0.050828 |
|  |  |  |  |  | F TEST | | 9.92E-07 |
|  |  |  |  |  | T TEST | | 0.000585 |
