# Supplementary material for: Visualization of stem cell activity in pancreatic cancer expansion by direct lineage tracing with live imaging
Source: eLife. 2021 Jan 4;10:e55117. doi: 10.7554/eLife.55117 (PMC7800378; doi:10.7554/eLife.55117)
Supplement: Figure 4—source data 1. — Measurement of EGFP+ area in liver tumor area derived from spleen-injected pancreatic ductal adenocarcinomas (PDACs) before (day 0) and 14 days after tamoxifen injection. Image J was used for the measurement. [file elife-55117-fig4-data1.docx]

**Figure 4-Source Data 1**

| day 0 (pixel) | EGFP^+^ area | Tumor area | %EGFP+ area | day 14 (pixel) | EGFP^+^ area | Tumor area | %EGFP^+^ area |
| --- | --- | --- | --- | --- | --- | --- | --- |
| LT 0_1 | 771 | 4138938 | 0.000186 | LT 14_1 | 517992 | 2326812 | 0.222619 |
| LT 0_2 | 930 | 6004047 | 0.000155 | LT 14_2 | 5970495 | 9346663 | 0.638784 |
| LT 0_3 | 403 | 3763310 | 0.000107 | LT 14_3 | 302430 | 1858517 | 0.162727 |
| LT 0_4 | 534 | 4233083 | 0.000126 | LT 14_4 | 886926 | 2668745 | 0.332338 |
| LT 0_5 | 732 | 3591571 | 0.000204 | LT 14_5 | 7588320 | 22589606 | 0.335921 |
| LT 0_6 | 653 | 10650451 | 0.000061 | LT 14_6 | 2803392 | 12120941 | 0.231285 |
|  |  | AVG | 0.000140 |  |  | AVG | 0.320612 |
|  |  | SD | 0.000053 |  |  | SD | 0.169773 |
|  |  | SE | 0.000022 |  |  | SE | 0.069310 |
|  |  |  |  |  | F TEST | | 3.14E-17 |
|  |  |  |  |  | T TEST | | 0.005716 |
