# Supplementary material for: Visualization of stem cell activity in pancreatic cancer expansion by direct lineage tracing with live imaging
Source: eLife. 2021 Jan 4;10:e55117. doi: 10.7554/eLife.55117 (PMC7800378; doi:10.7554/eLife.55117)
Supplement: Figure 6—source data 1. — Measured value of increasing curve of subcutaneous tumor derived from Dclk1+ PDACs cells sorted by FACS. [file elife-55117-fig6-data1.docx]

**Figure 6-Source Data 1**

| Size  (mm^3^) | 0w | 2w | 4W | 6w | 8w |
| --- | --- | --- | --- | --- | --- |
| Dclk1^+^_01 | 0 | 0 | 117.286 | 376.991 | 1017.876 |
| Dclk1^+^_02 | 0 | 0 | 75.398 | 209.440 | 670.206 |
| Dclk1^+^_03 | 0 | 0 | 100.531 | 261.799 | 804.248 |
|  |  |  |  |  |  |
| AVG | 0 | 0 | 97.738 | 282.743 | 830.777 |
| SD | 0 | 0 | 21.083 | 85.717 | 175.346 |
| SE | 0 | 0 | 12.172 | 49.489 | 101.236 |
| T TEST |  |  | 0.015 | 0.029 | 0.015 |
