# Supplementary material for: Visualization of stem cell activity in pancreatic cancer expansion by direct lineage tracing with live imaging
Source: eLife. 2021 Jan 4;10:e55117. doi: 10.7554/eLife.55117 (PMC7800378; doi:10.7554/eLife.55117)
Supplement: Supplementary file 1. [file elife-55117-supp1.docx]

**Supplementary File 1**

|  | Term | P-Value | Benjamini |
| --- | --- | --- | --- |
| 1 | cell adhesion | 6.60E-16 | 3.30E-12 |
| 2 | multicellular organism development | 6.50E-15 | 1.60E-11 |
| 3 | collagen fibril organization | 5.50E-12 | 9.10E-09 |
| 4 | extracellular matrix organization | 3.50E-10 | 4.30E-07 |
| 5 | ossification | 8.90E-10 | 8.70E-07 |
| 6 | angiogenesis | 9.70E-10 | 7.90E-07 |
| 7 | kidney development | 1.20E-09 | 8.50E-07 |
| 8 | positive regulation of cell migration | 3.70E-08 | 2.30E-05 |
| 9 | lung development | 5.70E-08 | 3.10E-05 |
| 10 | Wnt signaling pathway | 6.30E-08 | 3.10E-05 |
| 11 | axon guidance | 6.60E-08 | 2.90E-05 |
| 12 | negative regulation of cell proliferation | 8.10E-08 | 3.30E-05 |
| 13 | embryonic skeletal system morphogenesis | 4.90E-07 | 1.80E-04 |
| 14 | positive regulation of canonical Wnt signaling pathway | 7.90E-07 | 2.80E-04 |
| 15 | positive regulation of protein phosphorylation | 8.90E-07 | 2.90E-04 |
| 16 | heart development | 9.80E-07 | 3.00E-04 |
| 17 | palate development | 1.10E-06 | 3.20E-04 |
| 18 | embryonic skeletal system development | 1.80E-06 | 4.80E-04 |
| 19 | cartilage development | 2.10E-06 | 5.40E-04 |
| 20 | negative regulation of cell migration | 2.30E-06 | 5.50E-04 |
| 21 | embryonic digit morphogenesis | 2.60E-06 | 6.10E-04 |
| 22 | cellular response to transforming growth factor beta stimulus | 2.90E-06 | 6.50E-04 |
| 23 | negative regulation of Wnt signaling pathway | 3.20E-06 | 6.80E-04 |
| 24 | negative regulation of canonical Wnt signaling pathway | 3.40E-06 | 6.90E-04 |
| 25 | positive regulation of angiogenesis | 3.60E-06 | 7.10E-04 |
| 26 | positive regulation of osteoblast differentiation | 4.30E-06 | 8.20E-04 |
| 27 | vasculogenesis | 4.30E-06 | 8.20E-04 |
| 28 | vesicle-mediated transport | 1.60E-05 | 2.90E-03 |
| 29 | actomyosin structure organization | 2.10E-05 | 3.70E-03 |
| 30 | osteoblast differentiation | 2.50E-05 | 4.10E-03 |
| 31 | skeletal system development | 2.70E-05 | 4.30E-03 |
| 32 | middle ear morphogenesis | 4.00E-05 | 6.30E-03 |
| 33 | intramembranous ossification | 4.80E-05 | 7.30E-03 |
| 34 | anterior/posterior pattern specification | 6.00E-05 | 8.80E-03 |
| 35 | wound healing | 7.70E-05 | 1.10E-02 |
| 36 | response to hypoxia | 9.00E-05 | 1.30E-02 |
| 37 | positive regulation of epithelial cell proliferation | 9.10E-05 | 1.20E-02 |
| 38 | hippo signaling | 1.20E-04 | 1.50E-02 |
| 39 | positive regulation of bone mineralization | 1.30E-04 | 1.60E-02 |
| 40 | cell differentiation | 1.50E-04 | 1.90E-02 |
| 41 | response to peptide hormone | 1.70E-04 | 2.00E-02 |
| 42 | cellular response to platelet-derived growth factor stimulus | 1.80E-04 | 2.10E-02 |
| 43 | cell migration | 1.80E-04 | 2.10E-02 |
| 44 | glycosaminoglycan biosynthetic process | 1.90E-04 | 2.10E-02 |
| 45 | regulation of blood pressure | 2.10E-04 | 2.40E-02 |
| 46 | complement activation | 2.60E-04 | 2.80E-02 |
| 47 | nervous system development | 3.00E-04 | 3.10E-02 |
| 48 | negative regulation of neuron projection development | 3.20E-04 | 3.30E-02 |
| 49 | regulation of cell growth | 3.30E-04 | 3.30E-02 |
| 50 | positive regulation of smoothened signaling pathway | 3.60E-04 | 3.50E-02 |
| 51 | endochondral ossification | 3.60E-04 | 3.50E-02 |
| 52 | blood vessel development | 3.60E-04 | 3.50E-02 |
| 53 | smoothened signaling pathway | 3.60E-04 | 3.50E-02 |
| 54 | negative regulation of cell growth | 3.70E-04 | 3.50E-02 |
| 55 | mitral valve morphogenesis | 3.80E-04 | 3.50E-02 |
| 56 | response to oxidative stress | 4.20E-04 | 3.80E-02 |
| 57 | negative regulation of epithelial cell proliferation | 4.30E-04 | 3.90E-02 |
| 58 | digestive tract development | 4.80E-04 | 4.20E-02 |
| 59 | lung alveolus development | 5.00E-04 | 4.30E-02 |
| 60 | negative regulation of axon extension involved in axon guidance | 5.10E-04 | 4.30E-02 |
| 61 | negative chemotaxis | 5.10E-04 | 4.30E-02 |
| 62 | chaperone-mediated protein folding | 6.40E-04 | 5.30E-02 |
| 63 | odontogenesis | 7.10E-04 | 5.70E-02 |
| 64 | adrenal gland development | 7.10E-04 | 5.70E-02 |
| 65 | positive regulation of neuron projection development | 7.50E-04 | 5.90E-02 |
| 66 | wound healing, spreading of cells | 7.90E-04 | 6.10E-02 |
| 67 | negative regulation of fibroblast growth factor receptor signaling pathway | 7.90E-04 | 6.10E-02 |
| 68 | negative regulation of peptidase activity | 8.10E-04 | 6.20E-02 |
| 69 | positive regulation of smooth muscle cell migration | 9.20E-04 | 6.90E-02 |
| 70 | collagen biosynthetic process | 1.10E-03 | 8.30E-02 |
| 71 | substrate adhesion-dependent cell spreading | 1.20E-03 | 8.30E-02 |
| 72 | actin cytoskeleton organization | 1.20E-03 | 8.30E-02 |
| 73 | negative regulation of BMP signaling pathway | 1.20E-03 | 8.20E-02 |
| 74 | positive regulation of fibroblast proliferation | 1.20E-03 | 8.20E-02 |
| 75 | fat cell differentiation | 1.30E-03 | 9.00E-02 |
| 76 | transmembrane receptor protein tyrosine kinase signaling pathway | 1.40E-03 | 9.20E-02 |
| 77 | cellular response to BMP stimulus | 1.40E-03 | 9.20E-02 |
| 78 | protein heterotrimerization | 1.40E-03 | 9.30E-02 |
| 79 | positive regulation of endothelial cell migration | 1.40E-03 | 9.20E-02 |
| 80 | single organismal cell-cell adhesion | 1.50E-03 | 9.70E-02 |
| 81 | response to estradiol | 1.60E-03 | 9.70E-02 |
| 82 | negative regulation of transforming growth factor beta receptor signaling pathway | 1.60E-03 | 1.00E-01 |
| 83 | epithelial cell differentiation | 1.70E-03 | 1.00E-01 |
| 84 | positive regulation of BMP signaling pathway | 1.80E-03 | 1.00E-01 |
| 85 | positive regulation of cell-substrate adhesion | 1.80E-03 | 1.00E-01 |
| 86 | regulation of cell shape | 1.90E-03 | 1.10E-01 |
| 87 | transforming growth factor beta receptor signaling pathway | 2.00E-03 | 1.10E-01 |
| 88 | homophilic cell adhesion via plasma membrane adhesion molecules | 2.10E-03 | 1.20E-01 |
| 89 | bone development | 2.30E-03 | 1.30E-01 |
| 90 | protein transport | 2.30E-03 | 1.30E-01 |
| 91 | extracellular fibril organization | 2.40E-03 | 1.30E-01 |
| 92 | positive regulation of catenin import into nucleus | 2.40E-03 | 1.30E-01 |
| 93 | Type II pneumocyte differentiation | 2.50E-03 | 1.30E-01 |
| 94 | heart morphogenesis | 2.90E-03 | 1.50E-01 |
| 95 | female gonad development | 3.20E-03 | 1.60E-01 |
| 96 | ferric iron import into cell | 3.40E-03 | 1.70E-01 |
| 97 | Golgi organization | 3.50E-03 | 1.70E-01 |
| 98 | positive regulation of chondrocyte differentiation | 3.70E-03 | 1.80E-01 |
| 99 | positive regulation of cell growth involved in cardiac muscle cell development | 3.80E-03 | 1.80E-01 |
| 100 | negative regulation of vascular permeability | 3.80E-03 | 1.80E-01 |
