# Supplementary material for: Visualization of stem cell activity in pancreatic cancer expansion by direct lineage tracing with live imaging
Source: eLife. 2021 Jan 4;10:e55117. doi: 10.7554/eLife.55117 (PMC7800378; doi:10.7554/eLife.55117)
Supplement: Supplementary file 2. [file elife-55117-supp2.docx]

**Supplementary File 2**

|  | Term | P-Value | Benjamini |
| --- | --- | --- | --- |
| 1 | Focal adhesion | 7.10E-09 | 1.90E-06 |
| 2 | Axon guidance | 2.80E-08 | 3.80E-06 |
| 3 | PI3K-Akt signaling pathway | 3.60E-08 | 3.20E-06 |
| 4 | ECM-receptor interaction | 1.60E-07 | 1.10E-05 |
| 5 | Proteoglycans in cancer | 5.00E-06 | 2.70E-04 |
| 6 | Protein digestion and absorption | 8.80E-06 | 4.00E-04 |
| 7 | Hippo signaling pathway | 1.20E-05 | 4.60E-04 |
| 8 | Amoebiasis | 1.40E-05 | 4.70E-04 |
| 9 | Pathways in cancer | 4.40E-05 | 1.30E-03 |
| 10 | Rap1 signaling pathway | 9.70E-05 | 2.60E-03 |
| 11 | TGF-beta signaling pathway | 1.80E-04 | 4.40E-03 |
| 12 | Complement and coagulation cascades | 3.90E-04 | 8.70E-03 |
| 13 | Signaling pathways regulating pluripotency of stem cells | 6.30E-04 | 1.30E-02 |
| 14 | Drug metabolism - cytochrome P450 | 7.10E-04 | 1.40E-02 |
| 15 | Metabolism of xenobiotics by cytochrome P450 | 1.60E-03 | 2.80E-02 |
| 16 | Wnt signaling pathway | 2.00E-03 | 3.30E-02 |
| 17 | Pertussis | 2.40E-03 | 3.80E-02 |
| 18 | Glutathione metabolism | 3.40E-03 | 5.00E-02 |
| 19 | Amino sugar and nucleotide sugar metabolism | 3.90E-03 | 5.40E-02 |
| 20 | Protein processing in endoplasmic reticulum | 5.00E-03 | 6.60E-02 |
| 21 | Glycosaminoglycan biosynthesis - chondroitin sulfate / dermatan sulfate | 7.20E-03 | 8.90E-02 |
| 22 | Ras signaling pathway | 9.50E-03 | 1.10E-01 |
| 23 | Gap junction | 1.00E-02 | 1.20E-01 |
| 24 | Renin secretion | 1.10E-02 | 1.20E-01 |
| 25 | Hypertrophic cardiomyopathy (HCM) | 1.10E-02 | 1.20E-01 |
| 26 | Arginine and proline metabolism | 1.20E-02 | 1.10E-01 |
| 27 | cGMP-PKG signaling pathway | 1.30E-02 | 1.20E-01 |
| 28 | Histidine metabolism | 1.50E-02 | 1.30E-01 |
| 29 | Platelet activation | 1.50E-02 | 1.30E-01 |
| 30 | Tight junction | 1.60E-02 | 1.30E-01 |
| 31 | Dilated cardiomyopathy | 1.70E-02 | 1.40E-01 |
| 32 | Adrenergic signaling in cardiomyocytes | 1.80E-02 | 1.40E-01 |
| 33 | Chagas disease (American trypanosomiasis) | 2.40E-02 | 1.80E-01 |
| 34 | Glycine, serine and threonine metabolism | 2.60E-02 | 1.90E-01 |
| 35 | Melanoma | 2.60E-02 | 1.80E-01 |
| 36 | Adherens junction | 2.90E-02 | 2.00E-01 |
| 37 | Arrhythmogenic right ventricular cardiomyopathy (ARVC) | 3.90E-02 | 2.50E-01 |
| 38 | Protein export | 4.40E-02 | 2.70E-01 |
| 39 | Bacterial invasion of epithelial cells | 4.90E-02 | 2.90E-01 |
| 40 | Thyroid hormone synthesis | 5.10E-02 | 3.00E-01 |
| 41 | Basal cell carcinoma | 5.40E-02 | 3.10E-01 |
| 42 | Thyroid hormone signaling pathway | 5.40E-02 | 3.00E-01 |
| 43 | Valine, leucine and isoleucine degradation | 5.90E-02 | 3.20E-01 |
| 44 | Estrogen signaling pathway | 6.00E-02 | 3.20E-01 |
| 45 | Glycosaminoglycan biosynthesis - heparan sulfate / heparin | 6.20E-02 | 3.20E-01 |
| 46 | Melanogenesis | 6.50E-02 | 3.30E-01 |
| 47 | Vascular smooth muscle contraction | 6.90E-02 | 3.40E-01 |
| 48 | Chemical carcinogenesis | 7.40E-02 | 3.50E-01 |
| 49 | Cocaine addiction | 7.40E-02 | 3.40E-01 |
| 50 | Regulation of actin cytoskeleton | 7.60E-02 | 3.50E-01 |
| 51 | Cytokine-cytokine receptor interaction | 7.70E-02 | 3.40E-01 |
| 52 | Small cell lung cancer | 7.80E-02 | 3.40E-01 |
| 53 | Hematopoietic cell lineage | 8.40E-02 | 3.60E-01 |
| 54 | cAMP signaling pathway | 8.70E-02 | 3.70E-01 |
| 55 | Fatty acid metabolism | 8.90E-02 | 3.70E-01 |
| 56 | Metabolic pathways | 8.90E-02 | 3.60E-01 |
| 57 | Salivary secretion | 8.90E-02 | 3.60E-01 |
| 58 | Vasopressin-regulated water reabsorption | 9.20E-02 | 3.60E-01 |
