# Supplementary material for: Visualization of stem cell activity in pancreatic cancer expansion by direct lineage tracing with live imaging
Source: eLife. 2021 Jan 4;10:e55117. doi: 10.7554/eLife.55117 (PMC7800378; doi:10.7554/eLife.55117)
Supplement: Supplementary file 3. [file elife-55117-supp3.docx]

**Supplementary File 3**

**GSE16515 Gene Ontology, Biological Process**

|  | Term | P-Value | Benjamini |
| --- | --- | --- | --- |
| 1 | inflammatory response | 1.40E-21 | 5.80E-18 |
| 2 | immune response | 7.00E-18 | 1.50E-14 |
| 3 | T cell receptor signaling pathway | 1.50E-15 | 2.20E-12 |
| 4 | T cell costimulation | 9.90E-15 | 1.00E-11 |
| 5 | leukocyte migration | 5.20E-09 | 4.40E-06 |
| 6 | antigen processing and presentation of peptide or polysaccharide antigen via MHC class II | 6.60E-09 | 4.70E-06 |
| 7 | angiogenesis | 1.80E-08 | 1.10E-05 |
| 8 | cell surface receptor signaling pathway | 2.90E-08 | 1.60E-05 |
| 9 | signal transduction | 9.70E-08 | 4.50E-05 |
| 10 | T cell differentiation | 1.70E-07 | 7.40E-05 |
| 11 | adaptive immune response | 1.80E-07 | 7.00E-05 |
| 12 | T cell activation | 3.70E-07 | 1.30E-04 |
| 13 | innate immune response | 4.60E-07 | 1.50E-04 |
| 14 | cellular defense response | 1.00E-06 | 3.10E-04 |
| 15 | positive regulation of T cell proliferation | 2.90E-06 | 8.30E-04 |
| 16 | regulation of small GTPase mediated signal transduction | 6.70E-06 | 1.80E-03 |
| 17 | chemokine-mediated signaling pathway | 8.60E-06 | 2.10E-03 |
| 18 | interferon-gamma-mediated signaling pathway | 8.60E-06 | 2.10E-03 |
| 19 | positive regulation of interferon-gamma production | 8.80E-06 | 2.10E-03 |
| 20 | proteolysis involved in cellular protein catabolic process | 1.50E-05 | 3.40E-03 |
| 21 | regulation of cell shape | 1.50E-05 | 3.30E-03 |
| 22 | cell adhesion | 1.70E-05 | 3.50E-03 |
| 23 | positive regulation of GTPase activity | 1.70E-05 | 3.30E-03 |
| 24 | dendritic cell chemotaxis | 2.10E-05 | 3.90E-03 |
| 25 | positive regulation of ERK1 and ERK2 cascade | 2.20E-05 | 3.90E-03 |
| 26 | toll-like receptor signaling pathway | 2.50E-05 | 4.20E-03 |
| 27 | cellular response to lipopolysaccharide | 2.60E-05 | 4.30E-03 |
| 28 | regulation of immune response | 3.10E-05 | 4.90E-03 |
| 29 | negative regulation of interferon-gamma production | 3.60E-05 | 5.50E-03 |
| 30 | defense response to virus | 4.70E-05 | 6.80E-03 |
| 31 | positive regulation of inflammatory response | 5.00E-05 | 7.00E-03 |
| 32 | platelet degranulation | 5.70E-05 | 7.70E-03 |
| 33 | phagocytosis | 7.20E-05 | 9.50E-03 |
| 34 | antigen processing and presentation | 8.10E-05 | 1.00E-02 |
| 35 | negative regulation of angiogenesis | 8.50E-05 | 1.10E-02 |
| 36 | chemotaxis | 9.30E-05 | 1.10E-02 |
| 37 | vasculogenesis | 1.00E-04 | 1.20E-02 |
| 38 | apoptotic signaling pathway | 1.20E-04 | 1.40E-02 |
| 39 | negative regulation of tumor necrosis factor production | 1.30E-04 | 1.50E-02 |
| 40 | MyD88-dependent toll-like receptor signaling pathway | 1.80E-04 | 1.90E-02 |
| 41 | peptide antigen assembly with MHC class II protein complex | 1.90E-04 | 2.00E-02 |
| 42 | negative regulation of canonical Wnt signaling pathway | 2.30E-04 | 2.40E-02 |
| 43 | B cell receptor signaling pathway | 2.60E-04 | 2.60E-02 |
| 44 | calcium ion transport | 2.90E-04 | 2.80E-02 |
| 45 | B cell activation | 3.00E-04 | 2.90E-02 |
| 46 | cell chemotaxis | 5.30E-04 | 4.90E-02 |
| 47 | negative regulation of leukocyte apoptotic process | 5.40E-04 | 4.80E-02 |
| 48 | cell-cell signaling | 5.80E-04 | 5.00E-02 |
| 49 | neutrophil chemotaxis | 6.20E-04 | 5.30E-02 |
| 50 | positive regulation of interleukin-6 production | 6.70E-04 | 5.60E-02 |

**GSE16515 KEGG Pathway**

|  | Term | P-Value | Benjamini |
| --- | --- | --- | --- |
| 1 | Staphylococcus aureus infection | 3.00E-10 | 7.70E-08 |
| 2 | HTLV-I infection | 4.50E-10 | 5.90E-08 |
| 3 | Intestinal immune network for IgA production | 9.60E-10 | 8.40E-08 |
| 4 | Osteoclast differentiation | 6.80E-09 | 4.40E-07 |
| 5 | T cell receptor signaling pathway | 1.10E-08 | 5.50E-07 |
| 6 | Rheumatoid arthritis | 1.20E-08 | 5.30E-07 |
| 7 | Leishmaniasis | 1.60E-08 | 6.00E-07 |
| 8 | Asthma | 1.70E-08 | 5.70E-07 |
| 9 | Hematopoietic cell lineage | 4.50E-08 | 1.30E-06 |
| 10 | Allograft rejection | 5.60E-08 | 1.50E-06 |
| 11 | Graft-versus-host disease | 7.80E-08 | 1.90E-06 |
| 12 | Malaria | 1.10E-07 | 2.40E-06 |
| 13 | Cell adhesion molecules (CAMs) | 1.90E-07 | 3.80E-06 |
| 14 | Viral myocarditis | 2.40E-07 | 4.40E-06 |
| 15 | Natural killer cell mediated cytotoxicity | 2.50E-07 | 4.40E-06 |
| 16 | Phagosome | 6.90E-07 | 1.10E-05 |
| 17 | Cytokine-cytokine receptor interaction | 1.30E-06 | 2.00E-05 |
| 18 | Antigen processing and presentation | 1.40E-06 | 2.00E-05 |
| 19 | Autoimmune thyroid disease | 1.70E-06 | 2.30E-05 |
| 20 | Type I diabetes mellitus | 2.60E-06 | 3.30E-05 |
| 21 | Chemokine signaling pathway | 3.90E-06 | 4.80E-05 |
| 22 | Fc gamma R-mediated phagocytosis | 2.70E-05 | 3.20E-04 |
| 23 | Inflammatory bowel disease (IBD) | 3.10E-05 | 3.60E-04 |
| 24 | Primary immunodeficiency | 4.20E-05 | 4.60E-04 |
| 25 | Toll-like receptor signaling pathway | 8.40E-05 | 8.80E-04 |
| 26 | Toxoplasmosis | 1.50E-04 | 1.50E-03 |
| 27 | Tuberculosis | 1.60E-04 | 1.50E-03 |
| 28 | Epstein-Barr virus infection | 2.40E-04 | 2.30E-03 |
| 29 | Fc epsilon RI signaling pathway | 2.50E-04 | 2.30E-03 |
| 30 | B cell receptor signaling pathway | 3.00E-04 | 2.60E-03 |
| 31 | NF-kappa B signaling pathway | 4.60E-04 | 3.90E-03 |
| 32 | Influenza A | 6.40E-04 | 5.20E-03 |
| 33 | Ras signaling pathway | 1.10E-03 | 8.50E-03 |
| 34 | Rap1 signaling pathway | 1.30E-03 | 1.00E-02 |
| 35 | Regulation of lipolysis in adipocytes | 1.40E-03 | 1.00E-02 |
| 36 | Leukocyte transendothelial migration | 1.90E-03 | 1.40E-02 |
| 37 | Transcriptional misregulation in cancer | 3.60E-03 | 2.50E-02 |
| 38 | Herpes simplex infection | 5.90E-03 | 4.00E-02 |
| 39 | Pathogenic Escherichia coli infection | 6.50E-03 | 4.30E-02 |
| 40 | Chagas disease (American trypanosomiasis) | 8.40E-03 | 5.40E-02 |
| 41 | Systemic lupus erythematosus | 1.00E-02 | 6.40E-02 |
| 42 | MAPK signaling pathway | 3.20E-02 | 1.80E-01 |
| 43 | Aldosterone-regulated sodium reabsorption | 3.40E-02 | 1.90E-01 |
| 44 | mTOR signaling pathway | 4.00E-02 | 2.20E-01 |
| 45 | Jak-STAT signaling pathway | 4.20E-02 | 2.20E-01 |
| 46 | Bacterial invasion of epithelial cells | 4.60E-02 | 2.40E-01 |
| 47 | VEGF signaling pathway | 5.30E-02 | 2.60E-01 |
| 48 | Platelet activation | 5.60E-02 | 2.70E-01 |
| 49 | Proteasome | 5.90E-02 | 2.80E-01 |
| 50 | Chronic myeloid leukemia | 6.10E-02 | 2.80E-01 |

**GSE32676 Gene Ontology, Biological Process**

|  | Term | P-Value | Benjamini |
| --- | --- | --- | --- |
| 1 | inflammatory response | 3.70E-30 | 1.60E-26 |
| 2 | immune response | 2.10E-27 | 4.40E-24 |
| 3 | cell adhesion | 8.10E-17 | 1.60E-13 |
| 4 | extracellular matrix organization | 2.60E-14 | 2.70E-11 |
| 5 | signal transduction | 9.60E-14 | 8.10E-11 |
| 6 | regulation of immune response | 3.00E-12 | 2.10E-09 |
| 7 | cell chemotaxis | 4.40E-12 | 2.60E-09 |
| 8 | positive regulation of ERK1 and ERK2 cascade | 6.50E-12 | 3.40E-09 |
| 9 | phagocytosis, engulfment | 1.20E-11 | 5.50E-09 |
| 10 | T cell receptor signaling pathway | 2.70E-11 | 1.10E-08 |
| 11 | innate immune response | 5.20E-11 | 2.00E-08 |
| 12 | T cell costimulation | 7.10E-11 | 2.50E-08 |
| 13 | B cell receptor signaling pathway | 1.80E-10 | 5.70E-08 |
| 14 | positive regulation of T cell proliferation | 2.50E-10 | 7.60E-08 |
| 15 | leukocyte migration | 5.40E-10 | 1.50E-07 |
| 16 | positive regulation of inflammatory response | 3.10E-09 | 8.20E-07 |
| 17 | cell surface receptor signaling pathway | 3.60E-09 | 8.90E-07 |
| 18 | phagocytosis, recognition | 3.80E-09 | 8.90E-07 |
| 19 | antigen processing and presentation of peptide or polysaccharide antigen via MHC class II | 4.70E-09 | 1.00E-06 |
| 20 | angiogenesis | 6.80E-09 | 1.40E-06 |
| 21 | positive regulation of phosphatidylinositol 3-kinase signaling | 9.00E-09 | 1.80E-06 |
| 22 | chemokine-mediated signaling pathway | 9.60E-09 | 1.80E-06 |
| 23 | neutrophil chemotaxis | 1.20E-08 | 2.30E-06 |
| 24 | complement activation, classical pathway | 2.20E-08 | 3.80E-06 |
| 25 | complement activation | 2.70E-08 | 4.50E-06 |
| 26 | cellular response to interleukin-1 | 5.20E-08 | 8.40E-06 |
| 27 | positive regulation of nitric oxide biosynthetic process | 5.50E-08 | 8.70E-06 |
| 28 | cellular response to lipopolysaccharide | 1.00E-07 | 1.50E-05 |
| 29 | chemotaxis | 1.40E-07 | 2.10E-05 |
| 30 | positive regulation of B cell activation | 1.60E-07 | 2.20E-05 |
| 31 | regulation of cell shape | 2.60E-07 | 3.60E-05 |
| 32 | monocyte chemotaxis | 2.70E-07 | 3.60E-05 |
| 33 | positive regulation of angiogenesis | 5.50E-07 | 7.00E-05 |
| 34 | positive regulation of cell migration | 5.90E-07 | 7.30E-05 |
| 35 | negative regulation of angiogenesis | 6.30E-07 | 7.60E-05 |
| 36 | platelet degranulation | 8.30E-07 | 9.70E-05 |
| 37 | cellular response to interferon-gamma | 8.40E-07 | 9.60E-05 |
| 38 | interferon-gamma-mediated signaling pathway | 1.20E-06 | 1.40E-04 |
| 39 | positive regulation of tumor necrosis factor production | 1.40E-06 | 1.50E-04 |
| 40 | negative regulation of endopeptidase activity | 1.50E-06 | 1.60E-04 |
| 41 | negative regulation of cell proliferation | 1.90E-06 | 1.90E-04 |
| 42 | antigen processing and presentation | 2.50E-06 | 2.60E-04 |
| 43 | adaptive immune response | 2.90E-06 | 2.80E-04 |
| 44 | positive regulation of GTPase activity | 2.90E-06 | 2.80E-04 |
| 45 | positive regulation of peptidyl-tyrosine phosphorylation | 3.20E-06 | 3.00E-04 |
| 46 | T cell activation | 7.90E-06 | 7.30E-04 |
| 47 | cytokine production | 8.40E-06 | 7.60E-04 |
| 48 | positive regulation of monocyte chemotaxis | 9.40E-06 | 8.20E-04 |
| 49 | nervous system development | 1.00E-05 | 8.70E-04 |
| 50 | positive regulation of cytosolic calcium ion concentration | 1.10E-05 | 9.30E-04 |

**GSE32676 KEGG Pathway**

|  | Term | P-Value | Benjamini |
| --- | --- | --- | --- |
| 1 | Staphylococcus aureus infection | 1.10E-24 | 2.70E-22 |
| 2 | Leishmaniasis | 2.80E-16 | 4.30E-14 |
| 3 | Rheumatoid arthritis | 1.10E-14 | 9.20E-13 |
| 4 | Cell adhesion molecules (CAMs) | 7.60E-14 | 4.90E-12 |
| 5 | Phagosome | 2.80E-12 | 1.50E-10 |
| 6 | Chagas disease (American trypanosomiasis) | 8.10E-11 | 3.50E-09 |
| 7 | Chemokine signaling pathway | 1.10E-10 | 4.00E-09 |
| 8 | Complement and coagulation cascades | 2.00E-10 | 6.40E-09 |
| 9 | Graft-versus-host disease | 2.30E-10 | 6.70E-09 |
| 10 | Intestinal immune network for IgA production | 4.60E-10 | 1.20E-08 |
| 11 | Tuberculosis | 8.80E-10 | 2.10E-08 |
| 12 | Toxoplasmosis | 1.80E-09 | 4.00E-08 |
| 13 | Hematopoietic cell lineage | 2.00E-09 | 4.00E-08 |
| 14 | Type I diabetes mellitus | 3.00E-09 | 5.60E-08 |
| 15 | Inflammatory bowel disease (IBD) | 7.70E-09 | 1.30E-07 |
| 16 | Malaria | 8.30E-09 | 1.30E-07 |
| 17 | Antigen processing and presentation | 1.10E-08 | 1.70E-07 |
| 18 | HTLV-I infection | 2.00E-08 | 2.90E-07 |
| 19 | Asthma | 4.70E-08 | 6.50E-07 |
| 20 | Viral myocarditis | 1.40E-07 | 1.90E-06 |
| 21 | Allograft rejection | 1.60E-07 | 2.00E-06 |
| 22 | Osteoclast differentiation | 5.40E-07 | 6.40E-06 |
| 23 | Pertussis | 3.70E-06 | 4.20E-05 |
| 24 | Epstein-Barr virus infection | 4.10E-06 | 4.40E-05 |
| 25 | PI3K-Akt signaling pathway | 5.20E-06 | 5.40E-05 |
| 26 | Toll-like receptor signaling pathway | 9.20E-06 | 9.20E-05 |
| 27 | Leukocyte transendothelial migration | 1.30E-05 | 1.20E-04 |
| 28 | Autoimmune thyroid disease | 2.20E-05 | 2.00E-04 |
| 29 | Cytokine-cytokine receptor interaction | 2.30E-05 | 2.00E-04 |
| 30 | Systemic lupus erythematosus | 2.50E-05 | 2.20E-04 |
| 31 | NF-kappa B signaling pathway | 4.00E-05 | 3.40E-04 |
| 32 | Pathways in cancer | 6.10E-05 | 5.00E-04 |
| 33 | Measles | 6.10E-05 | 4.80E-04 |
| 34 | Influenza A | 8.00E-05 | 6.10E-04 |
| 35 | Amoebiasis | 8.90E-05 | 6.60E-04 |
| 36 | Herpes simplex infection | 2.10E-04 | 1.50E-03 |
| 37 | Proteoglycans in cancer | 2.20E-04 | 1.50E-03 |
| 38 | Rap1 signaling pathway | 2.60E-04 | 1.80E-03 |
| 39 | T cell receptor signaling pathway | 3.10E-04 | 2.10E-03 |
| 40 | Natural killer cell mediated cytotoxicity | 7.20E-04 | 4.70E-03 |
| 41 | Platelet activation | 7.20E-04 | 4.60E-03 |
| 42 | Transcriptional misregulation in cancer | 1.10E-03 | 6.80E-03 |
| 43 | ECM-receptor interaction | 1.10E-03 | 6.80E-03 |
| 44 | Sphingolipid signaling pathway | 1.40E-03 | 8.30E-03 |
| 45 | Prion diseases | 1.90E-03 | 1.10E-02 |
| 46 | HIF-1 signaling pathway | 3.40E-03 | 1.90E-02 |
| 47 | TNF signaling pathway | 4.70E-03 | 2.50E-02 |
| 48 | Small cell lung cancer | 6.00E-03 | 3.20E-02 |
| 49 | Ras signaling pathway | 6.90E-03 | 3.60E-02 |
| 50 | Prostate cancer | 8.30E-03 | 4.20E-02 |

**GSE36924 Gene Ontology, Biological Process**

|  | Term | P-Value | Benjamini |
| --- | --- | --- | --- |
| 1 | negative regulation of myotube differentiation | 0.0012 | 0.99 |
| 2 | protein autophosphorylation | 0.0018 | 0.97 |
| 3 | T cell costimulation | 0.011 | 1 |
| 4 | positive regulation of peptidyl-serine phosphorylation | 0.011 | 1 |
| 5 | neuron development | 0.012 | 1 |
| 6 | negative regulation of neuron apoptotic process | 0.012 | 1 |
| 7 | mitophagy in response to mitochondrial depolarization | 0.013 | 1 |
| 8 | microtubule-based movement | 0.015 | 1 |
| 9 | positive regulation of DNA-directed DNA polymerase activity | 0.015 | 1 |
| 10 | peptidyl-tyrosine dephosphorylation | 0.015 | 1 |
| 11 | positive regulation of peptidyl-tyrosine phosphorylation | 0.016 | 1 |
| 12 | positive regulation of cell migration | 0.017 | 1 |
| 13 | cellular response to estrogen stimulus | 0.018 | 1 |
| 14 | negative chemotaxis | 0.019 | 1 |
| 15 | macroautophagy | 0.021 | 1 |
| 16 | positive regulation of collateral sprouting | 0.023 | 1 |
| 17 | regulation of mitochondrial membrane potential | 0.024 | 1 |
| 18 | phosphatidylinositol-mediated signaling | 0.027 | 1 |
| 19 | peptidyl-serine phosphorylation | 0.027 | 1 |
| 20 | facial nerve structural organization | 0.032 | 1 |
| 21 | positive regulation of actin nucleation | 0.032 | 1 |
| 22 | mitochondrion transport along microtubule | 0.032 | 1 |
| 23 | muscle filament sliding | 0.033 | 1 |
| 24 | lipid metabolic process | 0.034 | 1 |
| 25 | T cell receptor signaling pathway | 0.035 | 1 |
| 26 | negative regulation of transforming growth factor beta receptor signaling pathway | 0.036 | 1 |
| 27 | ATP-dependent chromatin remodeling | 0.036 | 1 |
| 28 | regulation of JNK cascade | 0.037 | 1 |
| 29 | neurotrophin TRK receptor signaling pathway | 0.037 | 1 |
| 30 | anatomical structure morphogenesis | 0.038 | 1 |
| 31 | axon extension | 0.043 | 1 |
| 32 | axon development | 0.043 | 1 |
| 33 | negative regulation of apoptotic process | 0.045 | 1 |

**GSE36924 KEGG Pathway**

|  | Term | P-Value | Benjamini |
| --- | --- | --- | --- |
| 1 | MAPK signaling pathway | 0.0016 | 0.35 |
| 2 | T cell receptor signaling pathway | 0.0074 | 0.64 |
| 3 | Progesterone-mediated oocyte maturation | 0.011 | 0.65 |
| 4 | Signaling pathways regulating pluripotency of stem cells | 0.021 | 0.76 |
| 5 | Fc epsilon RI signaling pathway | 0.022 | 0.71 |
| 6 | Colorectal cancer | 0.029 | 0.74 |
| 7 | Non-alcoholic fatty liver disease (NAFLD) | 0.042 | 0.81 |
| 8 | Parkinson's disease | 0.044 | 0.78 |
| 9 | Epithelial cell signaling in Helicobacter pylori infection | 0.047 | 0.77 |

**TCGA_PAAD Gene Ontology, Biological Process**

|  | Term | P-Value | Benjamini |
| --- | --- | --- | --- |
| 1 | cell adhesion | 1.60E-13 | 1.50E-09 |
| 2 | angiogenesis | 6.40E-12 | 3.10E-08 |
| 3 | positive regulation of apoptotic process | 3.10E-09 | 9.90E-06 |
| 4 | leukocyte migration | 6.80E-09 | 1.70E-05 |
| 5 | signal transduction | 3.10E-08 | 6.00E-05 |
| 6 | positive regulation of GTPase activity | 7.10E-08 | 1.10E-04 |
| 7 | intracellular signal transduction | 1.90E-07 | 2.70E-04 |
| 8 | positive regulation of cytosolic calcium ion concentration | 3.10E-07 | 3.80E-04 |
| 9 | extracellular matrix organization | 3.40E-07 | 3.70E-04 |
| 10 | cell migration | 1.10E-06 | 1.00E-03 |
| 11 | protein phosphorylation | 2.80E-06 | 2.50E-03 |
| 12 | positive regulation of gene expression | 3.20E-06 | 2.60E-03 |
| 13 | negative regulation of transcription from RNA polymerase II promoter | 4.80E-06 | 3.60E-03 |
| 14 | peptidyl-tyrosine phosphorylation | 7.30E-06 | 5.00E-03 |
| 15 | negative regulation of angiogenesis | 8.10E-06 | 5.20E-03 |
| 16 | regulation of cardiac conduction | 8.50E-06 | 5.10E-03 |
| 17 | negative regulation of cell migration | 9.60E-06 | 5.50E-03 |
| 18 | positive regulation of transcription, DNA-templated | 1.20E-05 | 6.40E-03 |
| 19 | cell surface receptor signaling pathway | 1.80E-05 | 8.90E-03 |
| 20 | chemical synaptic transmission | 3.60E-05 | 1.70E-02 |
| 21 | sensory perception of pain | 5.00E-05 | 2.30E-02 |
| 22 | mitochondrial translational elongation | 5.20E-05 | 2.20E-02 |
| 23 | axonogenesis | 5.40E-05 | 2.30E-02 |
| 24 | positive regulation of peptidyl-tyrosine phosphorylation | 5.80E-05 | 2.30E-02 |
| 25 | neuron migration | 7.00E-05 | 2.70E-02 |
| 26 | positive regulation of phosphatidylinositol 3-kinase signaling | 7.80E-05 | 2.90E-02 |
| 27 | mitochondrial translational termination | 9.20E-05 | 3.20E-02 |
| 28 | T cell costimulation | 9.50E-05 | 3.20E-02 |
| 29 | cerebral cortex development | 1.00E-04 | 3.30E-02 |
| 30 | nervous system development | 1.00E-04 | 3.30E-02 |
| 31 | transmembrane receptor protein tyrosine kinase signaling pathway | 1.10E-04 | 3.20E-02 |
| 32 | negative regulation of gene expression | 1.80E-04 | 5.20E-02 |
| 33 | long-term memory | 1.80E-04 | 5.10E-02 |
| 34 | outflow tract morphogenesis | 1.90E-04 | 5.30E-02 |
| 35 | positive regulation of neuron projection development | 1.90E-04 | 5.20E-02 |
| 36 | cellular response to glucagon stimulus | 2.10E-04 | 5.40E-02 |
| 37 | adenylate cyclase-modulating G-protein coupled receptor signaling pathway | 2.10E-04 | 5.40E-02 |
| 38 | cellular response to hypoxia | 2.40E-04 | 5.90E-02 |
| 39 | negative regulation of cell growth | 2.50E-04 | 5.90E-02 |
| 40 | chloride transmembrane transport | 2.80E-04 | 6.50E-02 |
| 41 | axon guidance | 3.20E-04 | 7.40E-02 |
| 42 | semaphorin-plexin signaling pathway | 3.30E-04 | 7.40E-02 |
| 43 | positive regulation of excitatory postsynaptic potential | 3.50E-04 | 7.50E-02 |
| 44 | negative regulation of neuron apoptotic process | 3.50E-04 | 7.50E-02 |
| 45 | adenylate cyclase-inhibiting G-protein coupled receptor signaling pathway | 4.10E-04 | 8.40E-02 |
| 46 | protein autophosphorylation | 4.50E-04 | 9.10E-02 |
| 47 | T cell receptor signaling pathway | 4.60E-04 | 9.00E-02 |
| 48 | cell proliferation | 4.80E-04 | 9.30E-02 |
| 49 | positive regulation of Rho protein signal transduction | 5.00E-04 | 9.30E-02 |
| 50 | activation of phospholipase C activity | 5.00E-04 | 9.30E-02 |

**TCGA_PAAD KEGG Pathway**

|  | Term | P-Value | Benjamini |
| --- | --- | --- | --- |
| 1 | Cell adhesion molecules (CAMs) | 1.30E-10 | 3.90E-08 |
| 2 | Pathways in cancer | 2.70E-09 | 4.00E-07 |
| 3 | cGMP-PKG signaling pathway | 9.50E-08 | 9.40E-06 |
| 4 | Rap1 signaling pathway | 4.90E-07 | 3.60E-05 |
| 5 | Osteoclast differentiation | 6.50E-07 | 3.90E-05 |
| 6 | Focal adhesion | 7.50E-07 | 3.70E-05 |
| 7 | Cholinergic synapse | 9.20E-07 | 3.90E-05 |
| 8 | Chemokine signaling pathway | 1.40E-06 | 5.30E-05 |
| 9 | Retrograde endocannabinoid signaling | 1.80E-06 | 5.80E-05 |
| 10 | Ras signaling pathway | 3.00E-06 | 9.00E-05 |
| 11 | Proteoglycans in cancer | 4.10E-06 | 1.10E-04 |
| 12 | Adrenergic signaling in cardiomyocytes | 5.30E-06 | 1.30E-04 |
| 13 | Circadian entrainment | 5.90E-06 | 1.30E-04 |
| 14 | Axon guidance | 6.30E-06 | 1.30E-04 |
| 15 | Regulation of actin cytoskeleton | 2.80E-05 | 5.60E-04 |
| 16 | Alzheimer's disease | 2.80E-05 | 5.30E-04 |
| 17 | Platelet activation | 2.90E-05 | 5.00E-04 |
| 18 | Insulin secretion | 3.10E-05 | 5.10E-04 |
| 19 | PI3K-Akt signaling pathway | 3.60E-05 | 5.70E-04 |
| 20 | Calcium signaling pathway | 4.20E-05 | 6.20E-04 |
| 21 | T cell receptor signaling pathway | 4.50E-05 | 6.30E-04 |
| 22 | MAPK signaling pathway | 5.60E-05 | 7.60E-04 |
| 23 | Renal cell carcinoma | 7.80E-05 | 1.00E-03 |
| 24 | Leishmaniasis | 9.50E-05 | 1.20E-03 |
| 25 | Leukocyte transendothelial migration | 1.20E-04 | 1.40E-03 |
| 26 | Thyroid hormone signaling pathway | 1.20E-04 | 1.40E-03 |
| 27 | ECM-receptor interaction | 2.40E-04 | 2.80E-03 |
| 28 | Dilated cardiomyopathy | 2.70E-04 | 3.00E-03 |
| 29 | Amoebiasis | 3.90E-04 | 4.10E-03 |
| 30 | Vascular smooth muscle contraction | 6.00E-04 | 6.10E-03 |
| 31 | Glutamatergic synapse | 6.90E-04 | 6.80E-03 |
| 32 | Chagas disease (American trypanosomiasis) | 6.90E-04 | 6.60E-03 |
| 33 | Dopaminergic synapse | 8.50E-04 | 7.80E-03 |
| 34 | Staphylococcus aureus infection | 8.90E-04 | 8.00E-03 |
| 35 | Arrhythmogenic right ventricular cardiomyopathy (ARVC) | 9.90E-04 | 8.60E-03 |
| 36 | GABAergic synapse | 1.00E-03 | 8.50E-03 |
| 37 | Acute myeloid leukemia | 1.10E-03 | 9.40E-03 |
| 38 | Progesterone-mediated oocyte maturation | 1.20E-03 | 9.50E-03 |
| 39 | Neuroactive ligand-receptor interaction | 1.40E-03 | 1.10E-02 |
| 40 | Oxytocin signaling pathway | 1.50E-03 | 1.10E-02 |
| 41 | Adherens junction | 1.50E-03 | 1.10E-02 |
| 42 | Aldosterone synthesis and secretion | 1.60E-03 | 1.10E-02 |
| 43 | HIF-1 signaling pathway | 1.60E-03 | 1.10E-02 |
| 44 | Morphine addiction | 1.60E-03 | 1.10E-02 |
| 45 | Insulin resistance | 1.80E-03 | 1.20E-02 |
| 46 | Bacterial invasion of epithelial cells | 1.80E-03 | 1.20E-02 |
| 47 | Toxoplasmosis | 2.00E-03 | 1.30E-02 |
| 48 | HTLV-I infection | 2.00E-03 | 1.30E-02 |
| 49 | Renin secretion | 2.60E-03 | 1.60E-02 |
| 50 | Shigellosis | 2.60E-03 | 1.60E-02 |
